# Using Electronic Health Records to Enhance Lyme Disease Surveillance: Protocol for the SubLyme Network

**DOI:** 10.2196/94921

**Published:** 2026-07-08

**Authors:** Annemarie G Hirsch, Brian S Schwartz, Melissa N Poulsen, Anna M Schotthoefer, Maria E Sundaram, Jacob E Lemieux, Linden T Hu, Robert P Smith, Alexandra M Linz, Steph Battan-Wraith, Patrick K Mitchell, Cassandra A Hathaway, Jonathan S Pollak, Cara M Nordberg, Veronica Burkel, Courtney C Nawrocki Luskin, Kiersten J Kugeler, Alison F Hinckley, Sarah A Hook

**Affiliations:** 1 Department of Population Health Sciences Geisinger Danville, PA United States; 2 Department of Environmental Health and Engineering Bloomberg School of Public Health Johns Hopkins University Baltimore, MD United States; 3 Integrated Research and Development Laboratory Marshfield Clinic Research Institute Marshfield, WI United States; 4 Center for Clinical Epidemiology and Population Health Marshfield Clinic Research Institute Marshfield, WI United States; 5 Massachusetts General Hospital and Harvard Medical School Boston, MA United States; 6 Tufts School of Medicine Tufts University Boston, MA United States; 7 MaineHealth Institute for Research Portland, ME United States; 8 Westat Rockville, MD United States; 9 Division of Vector-Borne Diseases US Centers for Disease Control and Prevention Fort Collins, CO United States

**Keywords:** Lyme disease, surveillance, electronic health record, tick-borne disease, Borrelia burgdorferi

## Abstract

**Background:**

Lyme disease is the most common vector-borne illness in the United States. The limitations of traditional surveillance strategies for Lyme disease affect the ability to reliably track its burden and evaluate interventions. The US Centers for Disease Control and Prevention (CDC) established the Surveillance Based Lyme Disease Network (SubLyme) in September 2023 to strengthen Lyme disease surveillance and research using electronic health record (EHR) data.

**Objective:**

SubLyme has three primary objectives: (1) to establish and evaluate criteria for identifying Lyme disease cases in EHR data (ie, create computable phenotypes [CPs]) that can be scaled across diverse health systems, (2) to estimate Lyme disease incidence, and (3) to describe Lyme disease incidence by key demographics. Secondary objectives are to develop CPs that distinguish between acute and disseminated Lyme disease, identify clinical manifestations, and support future research efforts. This paper describes SubLyme, its structure, and its methods.

**Methods:**

SubLyme includes 5 health systems in 3 US regions with a high risk of Lyme disease: Geisinger, in Pennsylvania; Marshfield Clinic Health System, in Wisconsin; and Mass General Brigham, Tufts Medical Center, and MaineHealth in New England. The network is administered by a coordinating center (Westat) and the US CDC. SubLyme is evaluating the validity of EHR-based CP definitions for Lyme disease. CP performance is assessed by measuring sensitivity, specificity, positive predictive value, and negative predictive value against manually abstracted medical charts. Each site identified a cohort of patients with any Lyme disease element in their EHR (Lyme disease diagnosis code, Lyme disease laboratory test order, and Lyme-appropriate antibiotic order) during 2022 to 2023 and selected 500 charts for manual review as the gold standard against which CP performance was evaluated. SubLyme will use the Lyme disease CPs to generate incidence rates for Lyme disease overall and for various subgroups.

**Results:**

SubLyme identified 332,256 patients with at least 1 Lyme disease element in their record from more than 4.6 million patients. Of these patients, 55.6% (n=184,734) were female, 87.9% (n=292,053) were White, and 90.8% (n=301,688) were non-Hispanic. More than half of the patients only had a Lyme-appropriate medication order (n=177,425, 53.4%) and 35.8% (n=118,948) only had a Lyme disease test order. The most common combination was a medication order with a laboratory test order (n=22,926, 6.9%), followed by a combination of a diagnosis, test, and medication order (n=5316, 1.6%).

**Conclusions:**

SubLyme is well positioned to advance Lyme disease surveillance using EHR data across multiple health systems. The exploration of new surveillance methods in Lyme disease is critical as disease frequency increases and the geography expands. An EHR-based approach to surveillance has the potential to overcome challenges of current surveillance strategies and to accelerate Lyme disease research.

**International Registered Report Identifier (IRRID):**

DERR1-10.2196/94921

## Introduction

Lyme disease is the most common vector-borne illness in the United States, occurring in 2 major foci: the Northeast and mid-Atlantic regions and the upper Midwest [1]. Its incidence and geographic range are increasing, posing a significant public health burden [1]. Lyme disease surveillance data have been limited by the clinical and diagnostic complexities of the disease and the burden of disease reporting on clinicians and health departments [2]. A more robust disease surveillance strategy is essential to inform prevention, detection, and treatment strategies for populations and communities at greatest risk [2].

Lyme disease has been a nationally notifiable condition in the United States since 1991. Clinicians and laboratories report potential cases to state or local health officials, who classify these reports according to standardized surveillance case definitions developed by the Council of State and Territorial Epidemiologists (CSTE) [2]. Until recently, the CSTE case definitions required clinical details to determine case status, regardless of geography or frequency of the disease in the jurisdiction [2]. As case counts escalated in high-incidence regions, the workload of reviewing and validating clinical findings exceeded available resources, resulting in underestimates of Lyme disease incidence and variability in the quality and content of reported data across jurisdictions [2].

To address these limitations in systematic surveillance, the most recent CSTE case definition, updated in 2022, only requires a positive laboratory test in high-incidence states, with or without clinical confirmation, to classify someone as a probable case [3]. While it has likely improved consistency in reporting and reduced reporting burden, this update to the case definition could still be challenged by both overreporting and underreporting of disease. Using a definition based on serological testing alone might result in the inclusion of clinically incompatible or nonincident cases (ie, positive laboratory test result due to a previous infection) [2]. Conversely, it is probable that the new surveillance criteria do not capture cases for which laboratory evidence is not available, either because of insufficient time to build a detectable antibody response or because cases are presumptively treated without serological testing [2].

Electronic health record (EHR) data have the potential to overcome the challenges of traditional approaches to surveillance. Health system networks have been working together to develop EHR-based surveillance for chronic [4,5] and infectious diseases [6], with the goal of enriching traditional surveillance data with more timely and efficient approaches. EHR systems are widespread across the United States, with more than 90% of hospitals and nearly 90% of office-based physicians using an EHR system [7]. Clinical and demographic data are routinely collected and stored in EHR systems for clinical care purposes and can be used to produce timely surveillance estimates, without additional data collection or reporting. In EHR-based surveillance, case ascertainment is generally based on an automated EHR-based algorithm (ie, computable phenotype [CP]) rather than manual reporting and case classification methods. CPs are implemented using programming that can be quickly modified as needed, making this approach flexible compared to the more complex process of altering traditional surveillance strategies (eg, training of clinicians and health department personnel, revising data collection protocols, and undergoing consensus processes to changing CSTE case definitions) [4].

EHR systems have the potential to address many of the reasons for underreporting while reducing the labor needed for surveillance. Furthermore, the large population sizes captured by EHR systems have the potential to overcome sample size challenges of identifying and studying the less common, but sometimes severe, manifestations of disseminated Lyme disease (eg, carditis) [1]. Additionally, the longitudinal data in the EHR can be used to better understand the burden of posttreatment Lyme disease syndrome, and the large patient populations can be used to study outcomes in special populations (eg, pregnant women) and among demographic subgroups [8].

In 2022, the Centers for Disease Control and Prevention (CDC) issued a broad agency announcement requesting proposals to strengthen Lyme disease surveillance and research using EHR data. Awardees worked with the CDC to establish the Surveillance Based Lyme Disease Network (SubLyme). SubLyme will conduct network-wide Lyme disease surveillance in endemic regions, while informing future EHR-based surveillance strategies for Lyme disease at a national level. This paper describes SubLyme, its structure, and the methods that will be used to achieve the network objectives.

## Methods

### Study Design

SubLyme has three primary objectives: (1) to establish and evaluate criteria for identifying Lyme disease cases in EHR data (ie, create CPs) that can be scaled across diverse health systems, (2) to use the CPs to estimate incidence, and (3) to describe Lyme disease incidence by case demographics. A secondary objective is to develop alternative CPs that can distinguish between acute and disseminated Lyme disease and identify specific clinical manifestations (eg, dermatologic, neurological, joint, and cardiac) for routine estimates of disease severity. This systematic collection of standardized, real-time data on cases will enable researchers to monitor trends, identify risk factors, and evaluate the impact of prevention and treatment strategies. The protocol for achieving the primary aims is described in detail below.

### Ethical Considerations

Each SubLyme site, the coordinating center, and CDC received approval and waivers of consent from their local institutional review boards for this protocol: CDC (nonhuman subjects research determination), Geisinger (2016-0365), MaineHealth (2155117-7), Marshfield Clinic Research Institute (IRB-24-1487), Mass General Brigham (2023P002994), Tufts Health Sciences (STUDY00004649), and Westat (nonhuman subjects research determination).

### Study Setting and Population

**Figure 1:**
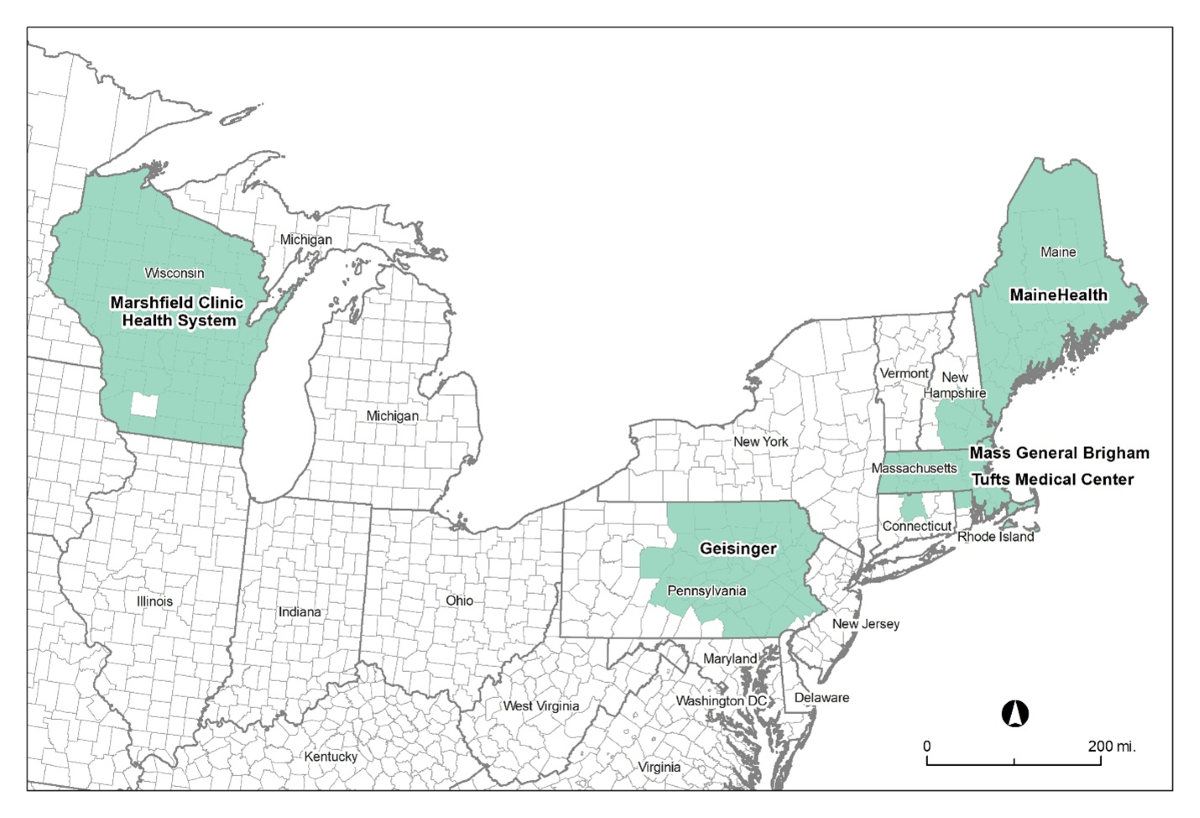
SubLyme sites and geographical coverage areas. Green indicates counties included in SubLyme surveillance of Lyme disease. The 2 unshaded Wisconsin counties are under surveillance but had no patients with evidence of Lyme disease in 2022 to 2023 (wide-net).

SubLyme includes EHR data from patients in 5 health systems in 3 US regions highly endemic to Lyme disease: Geisinger (Pennsylvania); Marshfield Clinic Health System (Wisconsin); and 3 health systems in New England—Mass General Brigham, Tufts Medical Center, and MaineHealth; the network is administered by a coordinating center (Westat) and directed by CDC [9]. The network covers 38 counties in Central and Northeastern Pennsylvania, 5 counties in Southern New Hampshire, 1 county each in Rhode Island and Connecticut, 70 counties in Wisconsin, and all counties in Maine and Massachusetts (Figure 1).

### Data Sources

EHR data from each of the 5 participating health systems will be the source of SubLyme data. Data elements will be extracted in the categories of demographics (eg, age, sex, race, ethnicity, and residential address), medication orders and medication indications, diagnoses, laboratory orders and results, clinical encounters (eg, outpatient, inpatient, and emergency department), and corresponding dates.

### Development of CPs

**Figure 2:**
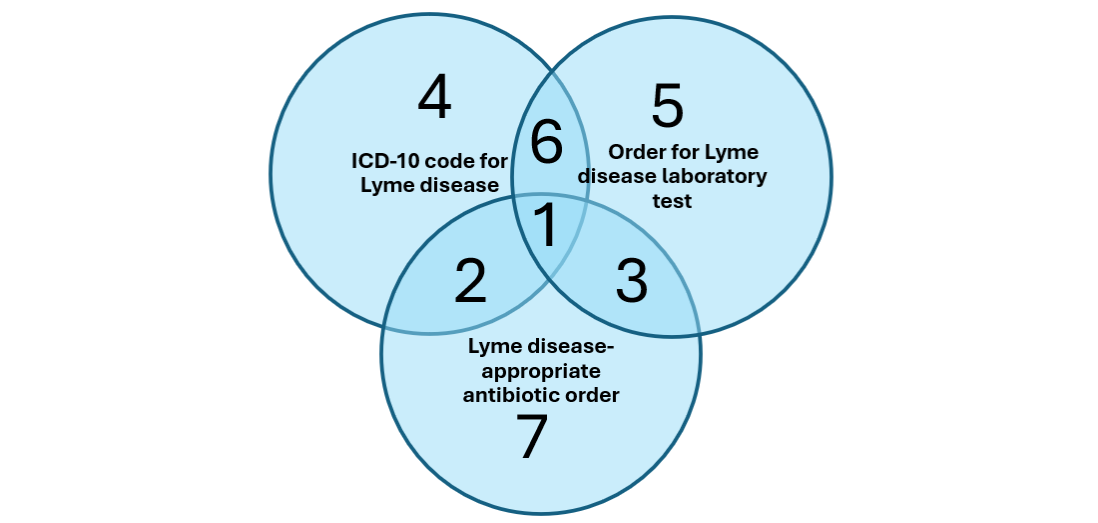
Seven mutually exclusive combinations of Lyme disease elements from the electronic health record: (1) diagnosis+laboratory test order+antibiotic order, (2) diagnosis+antibiotic order, (3) laboratory test order+antibiotic order, (4) diagnosis only, (5) laboratory test order only, (6) diagnosis and laboratory test order, and (7) antibiotic order only. ICD-10: International Classification of Diseases 10th Revision.

We are evaluating the Lyme disease CPs based on previously published methods of CP validation [10,11]. Each SubLyme site identified a Lyme disease wide-net cohort (those potentially suspected of having Lyme disease), defined as patients with any Lyme disease element in their EHR (*International Classification of Diseases-10* diagnosis codes for Lyme disease, Lyme disease laboratory test orders using Current Procedural Terminology codes, and Lyme disease–appropriate antibiotic orders) during 2022 to 2023 (Multimedia Appendix 1). This approach was designed to ensure high sensitivity. Thus, individuals not identified by these criteria were assumed not to have had Lyme disease in these years (ie, true negatives [TNs]). Then, patients in the wide net were categorized into 1 of 7 categories based on presence of different combinations of these Lyme disease elements within the calendar year (Figure 2). Next, sites created a reference panel for CP validation by randomly sampling 500 patients from each site in the wide-net population (2500 patients total), stratified by the 7 Lyme disease element categories.

### Chart Review

Sites conducted manual chart reviews of the reference panel sample, classifying patients into 1 of 5 categories to approximate CSTE 2017 criteria: likely Lyme disease (clinician observed rash and documented “erythema migrans” or “EM” or clinician observed or diagnosed Lyme arthritis, neuroborreliosis, or carditis and a positive laboratory test), possible Lyme disease (rash consistent with erythema migrans or nonspecific Lyme disease signs or symptoms with a positive, 2-tiered laboratory result for Lyme disease), not incident Lyme disease (evidence that patient presented with persistent symptoms from an earlier-occurring Lyme disease or the patient had a positive, 2-tiered laboratory result for Lyme disease in the absence of signs or symptoms), not Lyme disease, and insufficient information.

The chart review process was harmonized across SubLyme sites, facilitated by a standardized REDCap (version 17.1.2, Vanderbilt University) [12] abstraction form. Detailed instructions for chart reviewers were provided in a manual of procedures. Chart reviewers were trained during SubLyme meetings and internally at each site. Each site implemented interrater reliability assessments at the start of the study to calibrate chart abstraction and classification of case status. These efforts included independent review of selected records by multiple reviewers, reconciliation of discrepancies, and iterative refinement of the manual of procedures. The size and composition of chart review teams varied by site, based on staff availability. In the case of disagreements that could not be reconciled, teams consulted a physician for the final decision. Sites also brought challenging chart review cases to network-wide meetings for discussion and to reinforce consistent coding practices. Reviewers were not blinded to the CP status while conducting the chart review.

### Statistical Analysis

The network will evaluate the validity of EHR-based CP definitions, including both a priori*,* rule-based CPs (based on algorithms defined by subject matter experts) and machine learning–based CPs. For all CPs, sensitivity will be calculated as the true positives (TPs; classified as having Lyme disease by chart review and by CP) divided by all those classified as having Lyme disease by chart review (TP/[TP+false negatives]). Specificity will be calculated as the TNs (classified as not having Lyme disease by chart review and by CP) divided by all those classified as not having Lyme disease by chart review (TN/[TN+false positives]). Positive predictive value (PPV) will be calculated as the TPs divided by all those classified as having Lyme disease by CP (TP/[TP+false positives]). Finally, negative predictive value will be calculated as the TNs divided by all those classified as not having Lyme disease by CP (TN/[TN+false negatives]). We will create these metrics for 2 different definitions of TP, one that classifies only likely Lyme disease cases as positive for Lyme disease and one that classifies both likely and possible Lyme disease cases as positive for Lyme disease.

Measures will be primarily calculated combining all sites, with site-specific measures calculated as supplemental analysis. To account for stratified selection into the reference panel sample by Lyme disease element category, weighted measures will be calculated such that for each site, the proportion of total weights for observations in each category is equal to the proportion of wide-net events in that category. Final selection of the CPs for generating incidence estimates will be based on CP performance as well as other CDC standards for surveillance, including simplicity and timeliness [13].

### Machine Learning Analysis

Machine learning–based CPs will be developed using recursive partitioning trees and extensions thereof, including random forests and gradient boosted trees. Model predictors will be derived from structured EHR elements, including diagnosis codes, medication orders, and laboratory testing. Data will be split into separate training and testing subsets, with model performance assessed in the testing set.

### Incidence Estimation

Incidence calculations require estimation of the population at risk (“denominator”). Consistent with other EHR-based surveillance networks [4], each SubLyme site defined the denominator for incidence estimates using residential address and health care use history to identify the population from which Lyme disease status will likely be recorded in the health system’s EHR. Denominator approaches vary by SubLyme site based on health system–specific factors (eg, market share and geographic service area; Table 1). The numerator for incidence will be patients from the denominator populations who meet criteria for validated CPs. Each SubLyme site will provide unadjusted incidence rates overall and for demographic and geographic subgroups. The sensitivity and specificity of the CPs will be used to adjust for misclassification of cases and noncases through a Bayesian model. Specifically, the number of observed events meeting the CP will be modeled as a binomial distribution, with the probability of a positive classification defined as sensitivity×p_True Case_+specificity×(1−p_TrueCase_) The number of trials will equal the number of wide-net events, and prior distributions for sensitivity and specificity will be specified based on the values estimated during CP validation [14]. The 50th, 2.5th, and 97.5th percentiles of the resulting posterior distribution will be taken as the point estimate and 95% credible interval of the adjusted incidence numerator and adjusted incidence, calculated using the denominators described above.

**Table 1:** Denominator criteria by site.

Site	Denominator specifications
Geisinger	Live in 1 of 38 Pennsylvania counties with at least 1000 unique patient encounters in 2022 and 2023 (excluding Philadelphia and counties bordering Philadelphia) At least 1 clinical encounter (inpatient or outpatient) within the index year or in the 2 years prior
MaineHealth	At least 1 preventive visit or at least 2 evaluation and management encounters on different dates with a clinician type of MD^a^, DO^b^, NP^c^, PA^d^, or resident within the health system and within the past 5 years of January 1 of the year of interest
Marshfield Clinic Health System	At least 1 preventive visit or at least 2 evaluation and management encounters on different dates with a clinician type of MD, DO, NP, PA, or resident within the health system and within the past 5 years of July 1 of the year of interest
Mass General Brigham	Reside in 1 of the 14 counties in Massachusetts (all counties) or select bordering counties in New Hampshire (Belknap, Hillsborough, Merrimack, Rockingham, and Strafford), Rhode Island (Providence), or Connecticut (Hartford) Patients also have at least 2 evaluation and management encounters on different dates or at least 1 preventive visit with a clinician type of MD or DO, RN^e^, PA, NP, resident, or fellow within the health system and within the past 5 years of the index year
Tufts Medical Center	Individuals who have both a current address within the geographical region and who have encounters on at least 3 different dates during 5 years prior to the index year Exclude from the denominator any patients whose records have at least 1 of the following issues: (1) the patient record contains no valid birthdate and (2) the patient’s address does not contain both a county and zip code

^a^MD: doctor of medicine.

^b^DO: doctor of osteopathic medicine.

^c^NP: nurse practitioner.

^d^PA: physician assistant.

^e^RN: registered nurse.

### Missing Data

Missingness in EHR-based surveillance may be due to data quality issues in the extraction and transmission of data or could reflect data elements that are truly missing from a health system’s EHR due to clinical practices or documentation workflows [15]. We will assess data quality by evaluating the stability of missingness across time and consistency of missingness across sites. The coordinating center will work with sites to resolve missing data issues due to errors in data extraction or transmission. Consistent with other EHR-based surveillance networks, if it is determined that missingness correctly reflects the data from the EHR, no additional actions will be taken for incidence estimation [16]. As SubLyme moves into inferential analyses, more formal approaches to missingness will be considered, such as multiple imputation or inverse probability weighting.

### Data Sharing and Management

To facilitate network-wide analyses at the coordinating center, each site executed a data use agreement that permits the sharing of a limited dataset that includes chart review data and EHR data elements (eg, demographic characteristics, diagnoses, medication orders, laboratory orders, laboratory results, and relevant dates masked via date shifting) with the coordinating center. The coordinating center retains the data shared from each site. Only the site and the coordinating center have access to line-level data. Sites do not have access to line-level data from other sites. Data transfers between sites and the coordinating center are conducted via a secure file transfer protocol. The coordinating center manages these data centrally on a secure central platform with technical safeguards that include unique user identification and automatic log off. Sites have access to their own individual-level data and aggregate data from the network.

## Results

In preparation for the CP validation, each SubLyme site identified their wide-net population (Table 2). In total, the SubLyme wide-net population had 332,256 patients identified from more than 4.6 million patients. The wide-net cohort comprised 55.6% (n=184,734) female patients, 87.9% (n=292,053) White patients, and 90.8% (n=301,688) non-Hispanic patients. In total, 9.6% (n=31,896) of patients were aged 0 to 19 years, 20.2% (n=67,115) were aged 20 to 39 years, 46.2% (n=153,502) were aged 40 to 69 years, and 24% (n=79,741) were aged ≥70 years.

Among the wide-net cohort, more than half (n=177,092, 53.3%) only had a Lyme disease–appropriate medication order and 35.8% (n=118,948) only had a laboratory order for a Lyme disease test (Table 3). Among individuals with at least 2 Lyme disease elements, the most common was the combination of a Lyme disease–appropriate medication order with an order for a Lyme disease test (n=22,926, 6.9%), followed by the combination of a Lyme disease diagnosis, Lyme disease test order, and a corresponding medication order (n=5316, 1.6%). The least common combinations were those that did not include an order for a Lyme disease–appropriate medication.

**Table 2:** Characteristics of patients meeting the wide-net criteria in 2022 or 2023 (N=332,256)^a,b^.

Characteristics		Geisinger (n=97,022), n (%)	MaineHealth (n=51,148), n (%)	Marshfield Clinic Health System (n=20,470), n (%)	Mass General Brigham (n=132,693), n (%)	Tufts Medical Center (n=30,923), n (%)
**Age (years)**						
	0-19	12,246 (12.6)	4655 (9.1)	2518 (12.3)	10,338 (7.8)	2167 (7)
	20-39	19,576 (20.2)	8267 (16.2)	3338 (16.3)	28,912 (21.8)	6943 (22.4)
	40-69	44,636 (46)	21,864 (42.7)	9423 (46)	62,821 (47.3)	14,830 (47.9)
	≥70	20,564 (21.1)	16,362 (32)	5191 (25.4)	30,622 (23.1)	6983 (22.5)
**Sex**						
	Female	55,352 (57.1)	26,466 (51.7)	10,759 (52.6)	75,450 (56.9)	16,747 (54.1)
	Male	41,670 (43)	24,672 (48.2)	9710 (47.4)	57,220 (43.1)	14,166 (45.8)
	Unknown	0 (0)	0 (0)	0 (0)	0 (0)	10 (0)
**Race**						
	Asian or Pacific Island	1093 (1.1)	475 (0.9)	265 (1.3)	4698 (3.5)	2624 (8.5)
	Black	3317 (3.4)	1114 (2.2)	181 (0.9)	7078 (5.3)	1813 (5.8)
	Native American	280 (0.3)	179 (0.3)	292 (1.4)	382 (0.3)	0 (0)
	White	91,389 (94.2)	48,425 (94.7)	19,435 (94.9)	109,630 (82.6)	23,056 (74.5)
	Other or unknown	1558 (1.6)	955 (1.9)	446 (2.2)	10,950 (8.2)	3421 (11)
**Ethnicity**						
	Hispanic	4633 (4.8)	718 (1.4)	424 (2.1)	11,150 (8.4)	2969 (9.6)
	Non-Hispanic	90,729 (93.5)	50,102 (98)	19,347 (94.5)	113,465 (85.5)	27,954 (90.3)
	Other or unknown	1660 (1.7)	328 (0.6)	699 (3.4)	8078 (6.1)	0 (0)

^a^Lyme disease diagnosis code, Lyme disease test order, or medication order for a Lyme disease–appropriate antibiotic (oral doxycycline, oral amoxicillin, oral cefuroxime, or intravenous or injectable ceftriaxone) placed within 7 days of the first recorded Lyme disease–related element. Antibiotic orders with a documented indication other than Lyme disease were excluded. When no indication was recorded, antibiotic orders were excluded if a diagnosis for a condition commonly treated with antibiotics, other than Lyme disease, was documented within 7 days of the antibiotic order.

^b^Individuals who met Lyme disease wide-net criteria in both years were counted twice.

**Table 3:** Counts of wide-net cases by combination of Lyme disease (LD) element.

LD elements within calendar year	Geisinger, n (%)	MaineHealth, n (%)	Marshfield Clinic Health System, n (%)	Mass General Brigham, n (%)	Tufts Medical Center, n (%)
Medication^a,b^, LD diagnosis, and laboratory order	1515 (1.6)	1107 (2.2)	541 (2.6)	1717 (1.3)	292 (0.9)
Medication^a,b^ and LD diagnosis	1407 (1.5)	1066 (2.1)	535 (2.6)	1161 (0.9)	205 (0.6)
Medication^a,b^ and LD laboratory order	5122 (5.3)	5828 (11.4)	1458 (7.1)	9449 (7.1)	996 (3.2)
LD diagnosis	493 (0.5)	219 (0.4)	944 (4.6)	532 (0.4)	225 (0.7)
LD laboratory order	27,258 (28.1)	19,972 (39)	8646 (42.2)	53,392 (40.2)	9763 (31.5)
LD diagnosis and laboratory order	179 (0.18)	122 (0.2)	193 (0.9)	447 (0.3)	169 (0.5)
Medication^a^	61,048 (62.9)	22,834 (44.6)	8153 (39.8)	65,995 (49.7)	19,273 (62.3)

^a^Lyme disease–appropriate antibiotic order: doxycycline (oral), amoxicillin (oral), cefuroxime (oral), and ceftriaxone (intravenous or injection).

^b^Medication ordered within 7 days of the first recorded Lyme disease diagnosis or laboratory test order. Antibiotic orders with a documented indication other than Lyme disease were excluded. When no indication was recorded, antibiotic orders were excluded if a diagnosis for a condition commonly treated with antibiotics, other than Lyme disease, was documented within 7 days of the antibiotic order.

## Discussion

The exploration of novel surveillance methods for Lyme disease is increasingly important as disease incidence rises and the geographic range of the disease expands [1]. An EHR-based approach to surveillance has the potential to overcome existing barriers to case reporting and limitations of case criteria that impact the quality of current surveillance strategies and offer new opportunities for research. As a collaboration between CDC, large health care networks in endemic regions, and a central coordinating group, SubLyme is well positioned to advance Lyme disease surveillance and research through the use of EHR data. SubLyme will generate incidence estimates for regions of the United States with some of the highest Lyme disease burdens and will inform future strategies for enhancing nationwide surveillance using EHR data.

Targeted deployment of effective prevention, detection, and treatment strategies for disease requires high-quality surveillance data. There are emerging opportunities for prevention, including multiple Lyme disease vaccines at various stages of development, with clinical trials currently underway [2,17]. Enhanced surveillance will be essential for understanding Lyme disease vaccine uptake and for informing the design of studies to monitor vaccine safety and effectiveness. Over the last decade, EHR data have been increasingly used to monitor the safety and effectiveness of vaccines (eg, COVID-19, human papillomavirus, influenza, and pneumonia) [18,19] and will likely be critical in optimizing the impact of future Lyme disease vaccines and other novel preventive interventions.

Prior research demonstrates the feasibility of using EHR data to study Lyme disease [8,20-23]. SubLyme investigators have used EHR data to develop Lyme disease CPs [20,21] and to study individual and community-level risk factors for Lyme disease and Lyme disease stage and manifestation [8,23]. These studies have consistently reported higher estimates of Lyme disease incidence than more traditional surveillance approaches [8,23], in part because EHR-based estimates are not dependent on the time and effort of clinicians and health departments to report cases. By contrast, the EHR-based CPs used for generating these estimates also had moderate PPV and variable specificity, potentially resulting in inflated incidence estimates [20,21]. Nevertheless, EHR-based studies have resulted in generally similar demographic and seasonal patterns as those reported through traditional surveillance approaches over decades, lending confidence to these approaches [8,20].

EHR-based surveillance efforts face challenges. First, as noted above, Lyme disease CPs have been shown to have moderate performance in terms of PPV and specificity [20,21]. SubLyme will explore machine learning approaches to CPs and refine rule-based CP components (eg, laboratory results and antibiotics) to improve CP performance and the quality of incidence estimates. Second, EHR data are limited to individuals affiliated with the reporting health systems, and these populations may differ from the general population [24]. EHRs include data on the subset of the population that seeks care, potentially biasing EHRs toward greater coverage of women, children, and individuals who are more ill [4,24]. Third, the patient populations served by the SubLyme health systems are predominately White and non-Hispanic, potentially limiting generalizability to endemic regions of the country with different demographic profiles. However, the network includes thousands of individuals in different racial and ethnic groups, allowing for reporting of Lyme disease incidence in these subgroups. Additionally, CPs may perform differently in emerging or low-incidence states. Finally, manual review of EHR data is an imperfect gold standard for determining true Lyme disease case status due to the limitations of Lyme disease testing early in disease, inaccuracy of some diagnostic coding practices, and variability in clinician documentation.

SubLyme has important strengths. The health systems in the network are located across diverse geographic regions of the United States (Upper Midwest, Northeast, and Mid-Atlantic) and serve a range of community types, including urban and rural populations. Clinical manifestations of Lyme disease have been found to differ in regions of the Northeast and Midwest, attributed, in part, to differences in the genetic structure of the bacteria that cause Lyme disease in these regions [25,26]. Prior work has shown differences in Lyme disease risk and risk factors by degree of urbanicity [22]. The systems also differ in terms of their data access (eg, access to historic EHR data and diagnostic coding practices) and analytic infrastructure (eg, established data sharing practices and dedicated data analysts) that support EHR-based surveillance. Thus, the approach to surveillance will be designed to be flexible and generalizable to a broad range of health systems. SubLyme is multidisciplinary, with experts in epidemiology, infectious disease, vector-borne disease, molecular biology, disease surveillance, Lyme disease, computational biology, and EHR-based epidemiology, with diverse perspectives informing well-rounded surveillance strategies. Finally, in addition to advancing surveillance methodology, SubLyme will identify large cohorts of well-characterized individuals with Lyme disease for whom longitudinal EHR data are available across the network. The data available on these cohorts can inform future research in a variety of areas, including Lyme disease staging and manifestations; posttreatment Lyme disease syndrome; and, as previously noted, vaccine safety and effectiveness.

## Appendix

Multimedia Appendix 1 Lyme disease wide-net cohort specifications.

Multimedia Appendix 2 Peer-reviewer report from the US Centers for Disease Control and Prevention (CDC).

## Abbreviations

CDC: Centers for Disease Control and Prevention
CP: computable phenotype
CSTE: Council of State and Territorial Epidemiologists
EHR: electronic health record
PPV: positive predictive value
SubLyme: Surveillance Based Lyme Disease Network
TN: true negative
TP: true positive

## Data Availability

SubLyme data are derived from electronic health records and contain protected health information. Due to patient privacy considerations, these data are not publicly available. Deidentified or limited datasets may be made available upon request, subject to review and approval by the institutional review boards of participating sites and in compliance with data use agreements. Researchers interested in requesting access to data should contact Westat.
