# Supplementary material for: Using Electronic Health Records to Enhance Lyme Disease Surveillance: Protocol for the SubLyme Network
Source: JMIR Res Protoc. 2026 Jul 8;15:e94921. doi: 10.2196/94921 (PMC13392523; doi:10.2196/94921)
Supplement: Multimedia Appendix 1 [file resprot_v15i1e94921_app1.docx]

**Supplemental 1.** Specification of Data Elements for Eligibility

**ICD-10 Codes**

| **ICD-10 Code** | **Code Definition** |
| --- | --- |
| A69.2 | Lyme disease |
| A69.20 | Lyme disease unspecified |
| A69.21 | Meningitis due to Lyme disease |
| A69.22 | Other neurologic disorders in Lyme disease |
| A69.23 | Arthritis due to Lyme disease |
| A69.29 | Other conditions associated with Lyme disease |

**Testing**

| **Test Code** | **Code Type** | **Test Name** |
| --- | --- | --- |
| 86618 | CPT | Lyme disease antibody screen with reflex to confirmation |
| 86617 | CPT | Lyme disease antibodies (IgG, IgM), Immunoblot |
| 87475 | CPT | Infectious agent detection by nucleic acid (DNA or RNA); Borrelia burgdorferi, direct probe technique |
| 87476 | CPT | Infectious agent detection by nucleic acid (DNA or RNA); Borrelia burgdorferi, amplified probe technique |

**Medication Orders**

| **Medication** | **Route** | **Exclusions** |
| --- | --- | --- |
| Amoxicillin | Oral |  |
| Azithromycin | oral | Exclude Z-Pak |
| Cefotaxime | IV |  |
| Ceftriaxone | IV | Exclude intramuscular route |
| Cefuroxime | oral |  |
| Clarithromycin | oral |  |
| Doxycycline | oral | Exclude single dose |
| Erythromycin | oral |  |
| Penicillin G | IV |  |
| Exclude antibiotics with an indication for something other than Lyme disease. When antibiotics do not include an indication, exclude antibiotics if there is a diagnosis for conditions other than Lyme disease that are commonly treated with antibiotics within 7 days of the antibiotic order. | | |
