# Supplementary material for: Using Electronic Health Records to Enhance Lyme Disease Surveillance: Protocol for the SubLyme Network
Source: JMIR Res Protoc. 2026 Jul 8;15:e94921. doi: 10.2196/94921 (PMC13392523; doi:10.2196/94921)
Supplement: Multimedia Appendix 2 [file resprot_v15i1e94921_app2.docx]

### SCORING SHEETS FOR INDIVIDUAL EVALUATORS

RFQ Number: 75D301-24-R-72992 Titled: SubLyme Network: The Geisinger-Johns Hopkins Site

OFFEROR NAME: Geisinger-Johns Hopkins Bloomberg School of Public Health (collaboration)

PANEL MEMBER NAME: _________Alison Hinckley, PhD___________________________

**1.** **Technical Approach and Understanding** – **Technically Acceptable**

The offeror represents a large health system in a high incidence area and has been participating in the SubLyme program for the past year. They describe a reasonable approach to all 3 primary objectives, in line with what has been conceived of within the BAA-funded SubLyme network. They also have thorough plans listed for secondary objectives. Note, prior experience with CDC-funded networks (e.g. DiCAYA) has been helpful to move forward expectations and protocols for surveillance estimates. In addition, plans to translate a Lyme disease CP using a Common Data Element (CDE) model are also nicely described and supported.

Offeror does propose to leverage EHR-based identification of patients for other tickborne diseases, which is of interest. Offeror also expresses a willingness to work with CDC and other regional parties to maximize research efficiency and impact. This is important given CDCs stated aim to establish surveillance and research platforms in geographically distinct high incidence locations that generate comparable data and consistent analyses. Particular strengths of this application include plans to analyze and inform surveillance models for distinct clinical manifestations and post-treatment Lyme disease syndrome.

**2.** **Staffing Plan**– **Technically Acceptable**

The leadership team is comprised of experienced individuals with appropriate skills. The technical team includes oversight by two individuals with extensive relevant experience, with support from data programmer/analysts, program/project managers. Others, including a pediatric infectious disease specialist, can be brought on depending upon project progress and changing scope. which could all be useful in future years of the surveillance network activities. The skills and expertise of the other-mentioned support staff (e.g. research assistants) have been appropriately updated in the amended SOW to reflect more appropriate distributions in first and option years.

**3.** **Management Approach** – **Technically Acceptable**

The management approach is acceptable for including experienced leads from both collaborating sites as well as appropriately skilled technical support staff.

**4.** **Similar Experience** – **Technically Acceptable**

Given that this offerer was funded through the BAA process in 2023/2024 for an electronic health record surveillance project for Lyme disease, they already have one year of completely relevant experience towards development of this research network under a common protocol. In addition, they have other relevant CDC network experience that enhances their contribution to SubLyme.

## Overall Technical Evaluation Criteria: Technically Acceptable

This application is technically adequate for technical approach and staffing. The offerer included good detail regarding next step plans for a network approach and potential for option year efforts and associated updates to staffing plans.

Alison Hinckley

07/24/24


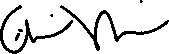


_______________________________

Panel Member Signatures

### SCORING SHEETS FOR INDIVIDUAL EVALUATORS

RFQ Number: 75D301-24-R-72992 Titled: SubLyme Network Year 2: The Geisinger-Johns Hopkins Site

OFFEROR NAME: Geisinger

PANEL MEMBER NAME: _Sarah Hook___________________________________

**1.** **Technical Approach and Understanding** – Technically Acceptable

Overall, the offeror’s proposal is well written and fully responsive to the solicitation. The proposal has major strengths in demonstrating understanding of the solicitation’s goals and objectives, as well as providing detailed plans for special studies proposed (or why some special studies were not proposed).

**2.** **Staffing Plan**– Technically Acceptable

The offerors adequately address our few clarifying questions, specifically they have now linked staffing roles to specific solicitation objectives in their responses (also included in their revised budget).

**3.** **Management Approach** – Technically Acceptable

The management approach is technically acceptable; it is sufficiently described and should be able to adequately address management issues, should they arise.

**4.** **Similar Experience** – Technically Acceptable

The offerors do a superb job describing their efforts as part of the SubLyme network to date. Not only do they have experience and strong performance in the SubLyme network (that justifies their continued involvement, per this solicitation), but they also provide a strong description of other related work querying electronic health records.

## Overall Technical Evaluation Criteria: Technically Acceptable


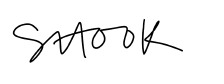


_______________________________July 22, 2024

Panel Member Signature
